# Effects of an integrative treatment, therapeutic acupuncture and conventional treatment in alleviating psychological distress in primary care patients - a pragmatic randomized controlled trial

**DOI:** 10.1186/1472-6882-13-308

**Published:** 2013-11-07

**Authors:** Tina Arvidsdotter, Bertil Marklund, Charles Taft

**Affiliations:** 1 Institute of Health and Care Sciences, Sahlgrenska Academy, University of Gothenburg, Gothenburg, Sweden; 2 Department of Primary Health Care, University of Gothenburg, Gothenburg, Sweden; 3 Research and Development Center Fyrbodal, Vänersborg, Sweden

**Keywords:** Acupuncture, Anxiety, Depression, Holistic, Integrative treatment, Primary care, Psychological distress, Salutogenesis

## Abstract

**Background:**

To evaluate and compare effects of an integrative treatment (IT), therapeutic acupuncture (TA), and conventional treatment (CT) in alleviating symptoms of anxiety and depression in psychologically distressed primary care patients.

**Methods:**

An open, pragmatic randomized controlled trial comparing the three treatment regimens at four and eight weeks after treatment. The study sample consisted of 120 adults (40 per treatment arm) aged 20 to 55 years referred from four different primary health care centres in western Sweden for psychological distress. Psychological distress was evaluated at baseline, and after 4 and 8 weeks of treatment using the Hospital Anxiety and Depression scale (HAD). Treatment sessions lasted about 60 minutes in IT and 45 minutes in TA.

**Results:**

No baseline differences were found between groups on HAD depression or anxiety. HAD anxiety and depression decreased significantly more in the IT and TA groups than in the CT group both after 4 and 8 weeks of treatment, but not between IT and TA. Improvements in the TA and IT groups were large and clinically significant, whereas CT effects were small and clinically non-significant.

**Conclusions:**

Both IT and TA appear to be beneficial in reducing anxiety and depression in primary care patients referred for psychological distress, whereas CT does not. These results need to be confirmed in larger, longer-term studies addressing potentially confounding design issues in the present study.

**Trial registration:**

ISRCTN trial number NCT01631500.

## Background

Psychological distress refers to a state of general emotional discomfort in response to a stressor or demand [1] and is typically characterized by symptoms of depression and anxiety [2-4]. These symptoms often coexist [5] and co-occur with common somatic complaints [6] e.g., insomnia, headaches and fatigue; a wide range of chronic conditions [7], as well as with medically unexplained syndromes [8] e.g., IBS and fibromyalgia. Psychological distress is associated with disability [9] and poor adherence to treatment protocols [10]. Moreover, it is also associated with premature mortality among primary care patients [11] and an elevated risk of, for example, cardiovascular disease [12] and cancer [13].

With prevalence rates of 10-38%, psychological distress is a common mental health problem in the community [14-17]. Although many who experience psychological distress never seek professional help [18,19], most of those who do are treated within primary care [20]. General practitioners estimate that about 25% of their patients have mental health problems [21], however, as psychological distress often goes undetected in primary care settings [22,23] its prevalence may be considerably higher.

Current guidance suggests watchful waiting, pharmacological and non-pharmacological treatment and referral to specialist care as possible strategies for treating psychological distress, depending on the severity of symptoms, level of functional impairment, as well as patient preferences [24,25]. Watchful waiting accompanied by general advice may be recommended in mild cases, given that many patients experience spontaneous resolution of their symptoms over time [26]. Nonetheless, patients often consider this strategy to be unhelpful and prefer active treatment [27]. Psychotropic medication is recommended for moderate-to-severe cases [24,25], although their use even in severe cases is debated [28]. Up to one-fifth of patients have been found to consider medication unbeneficial [27] and many patients prefer not to take such medication [29]. A wide variety of non-pharmacological interventions, including psychological, behavioral and psychosocial therapies, may be offered in primary care and there is some evidence for their efficacy, particularly cognitive behavioral therapy [30].

The use of complementary and alternative medicine (CAM) modalities for treating psychological distress is substantial [31] and it has been suggested that people with this condition are more likely to use CAM therapies than conventional medical or mental health treatments [32]. One of the most widely used CAM therapies is acupuncture [33]. Acupuncture has been shown to reduce psychological distress in many studies and four systematic reviews, including one from the Cochrane Collaboration, concluded that although results to date have been mixed and despite methodological deficiencies, acupuncture appears to be a promising treatment option for psychological distress [34-37]. For example, a moderate-large effect size (.73) between manual acupuncture versus waiting list in reducing depression was reported in a recent Cochrane review [37]. The mechanisms underlying the effects of acupuncture on psychological distress are not fully understood. Acupuncture may influence neurotransmitter and hormonal pathways underlying emotional states. For example, acupuncture stimulates secretion of serotonin and norepinephrine [38], two neurotransmitters presumed to be associated with depression [39]. Acupuncture may also stimulate sensory nerves, induce the release of endogenous opioids and modulate the autonomic nervous system [40] and thereby influence mood and increase the patient’s sense of well-being.

In this study, we hypothesize that the potential therapeutic effects of acupuncture in alleviating psychological distress may be strengthened by integrating acupuncture [41] with non-directive, salutogenic counseling [42] in a person centered approach [43,44] to help patients gain insight into obstacles to and resources for managing their condition, combined with supportive advice about lifestyle, coping strategies and behavior changes to promote health.

### Aim

The aim was to evaluate the relative efficacy of conventional treatment (CT) vs. therapeutic acupuncture (TA) vs. an integrative treatment (IT) in reducing psychological distress in primary care patients.

## Methods

### Design and setting

This longitudinal open, pragmatic randomized controlled study comparing CT, TA and IT was conducted at four primary health care centers in western Sweden during the period 2010–2011. All 10 centers in the region were contacted and four agreed to participate in the study. Two of the centers (one private and one public) were located in small towns (<15,000 inhabitants) and two centers (one private and one public) were in medium-sized towns (15,000-40,000 inhabitants). The study was approved by the Regional Ethical Review Board, Gothenburg Sweden (Dnr: 365–08).

### Participants

The target population comprised primary care patients 20–55 years old presenting with complaints of psychological distress, such as worry, anxiety, depression, sleep disturbances, fatigue, headache or somatic pain. Exclusion criteria were full sick leave >2.5 years, pregnancy, cancer, personality disorders, substance or alcohol use disorders and severe depression.

Patients were initially recruited through referrals from physicians; however, to hasten inclusion, referrals were later accepted from other healthcare professionals and in some cases from the patients themselves. All participants sought treatment for their complaints of PD. In total, 150 patients were referred by healthcare professionals (doctors, nurses, psychotherapists and welfare officers) and four were self-referrals, all of them via their health care center. Eligible referrals were contacted by phone and informed about the study. Consenting patients were scheduled for a visit at their primary health care center. At the visit the patients’ medical histories were reviewed by a research nurse and in semi-structured interviews they were asked to describe their illness experience, including symptoms and their consequences on daily life, etc. The visits lasted approximately 60 minutes. Based on information derived during the visit an additional 34 patients failed to meet inclusion criteria and were excluded. Hence, 120 persons (101 women) were included in the study (Table 1). The final sample was subsequently mailed written information about the study, along with a written consent form and the first set of questionnaires, which they were asked to complete at home and return at their next visit.

**Table 1:** Patient sociodemographic characteristics

**Variable**	**IT n = 40**	**TA n = 40**	**CT n = 40**
Sex: female	34 (85%)	32 (80%)	35 (88%)
Mean age (SD)	41	41	40
Education: High school	26 (65%)	28 (70%)	23 (58%)

Integrative Treatment, IT, Therapeutic acupuncture, TA, Conventional treatment, CT.

Thirty percent of the patient sample had a primary diagnosis of depression, 20% had anxiety or panic disorders, 20% had severe stress, 20% somatic symptoms/ pain (including irritable bowel syndrome, fibromyalgia, migraine, fatigue) and 10% sleep disorders. About 80% had secondary diagnoses including one or more of the above primary diagnoses.

### Interventions

All patients who had been prescribed medication for depression, anxiety, sleep disturbances or pain prior to study inclusion were advised to continue their medication regimens as usual. However, patients in the TA and IT groups were asked not to begin psychological treatments or physiotherapy during the study period. In both groups; TA and IT, the participants were treated by the same therapist. The therapist had nine years of experience of salutogenic dialogue inspired by Antonovsky’s sense of coherence theory [42], which is included in nursing education in Sweden. The salutogenic dialogue included three components: comprehensibility (cognitive), manageability (behavioral), and meaningfulness (motivational) and reflects a person’s capacity to manage stressful situations and stay well [45].The therapist also had nine years of clinical experience as a TCM acupuncturist.

#### Therapeutic acupuncture

Therapeutic acupuncture (TA) [41] was performed once a week for eight consecutive weeks by a certified TCM acupuncturist. Each session lasted approximately 45 minutes. Selection of acupuncture points was based on clinical RCTs and TCM (Table 2).

**Table 2:** **Acupuncture protocol in accordance with STandards for Reporting Interventions in Controlled Trials of Acupuncture, STRICTA **[46]

**Item**	**Intervention**	**Details**
1	Integrative treatment , IT	Therapeutic acupuncture treatment of sedative and restorative acupuncture points [41] integrated with structured salutogenic [42] dialogue [43,44].
2	Needling details	Points used; Gv 20, Cv 6, Pc 6, Ht 7, Co 4, Lr 3, Sp 6, St 36 and accipoints. Uni-or bilateral 2–10 needles each time Depths of insertion: 0.5-1.5 cm Responses elicited: de qi Needle stimulation: manual Needle retention time: 20–30 minutes Needle type: Sterile disposable needles brand Vinco of stainless steel, size 0.30x25 mm
3	Integrative treatment, IT	8 treatment sessions once a week for 8 weeks 60 minutes session
4	Co-interventions	Other interventions; exercise and relaxation with briefing techniques
5	Practitioner background	Research nurse, trained in salutogenic dialogue, basic psychotherapy education, TCM acupuncturist, Education at Swedish TCM school. 9 years of clinical acupuncture experience
6	Control intervention 1 Therapeutic acupuncture, TA	Therapeutic acupuncture with unstructured salutogenic dialogue, once a week for 8 weeks. 45 minutes session
6	Control intervention 2 Conventional treatment, CT	Conventional treatment in primary care for 8 weeks

Needles were inserted gently but firmly until a de qi sensation (feeling of numbness, fullness, or soreness) was experienced by the participant. At least two needles were used and when necessary additional needles (maximum 12 needles) were inserted until de qi was achieved. Treatment lasted 20–30 minutes. While performing TA, the acupuncturist conversed freely with the patient about his/her condition and suggested various lifestyle changes and relaxation methods for the patient to practice at home.

#### Integrative treatment

The Integrative treatment (IT) combined TA with a salutogenic dialogue. Inspired by Antonovsky’s salutogenic model [42], the dialogue focused on the patient’s understanding of meaning ascribed to and resources for managing his/her condition. The aim was to help the patients to become aware of and mobilize their strengths and potentials for coping with their condition and emphasized a person-centred perspective [44,47,48]. The dialogue was open, supportive, exploratory and reflective, and concentrated on four crucial areas of life: inner feelings, personal relations, everyday activities (diet, exercise, relaxation, sleep habits) and existential issues. It was conducted in an atmosphere of trust, respect, empathy and genuineness [43], with the aim to strengthen the therapeutic alliance [48].

IT was performed once a week for eight consecutive weeks by the same therapist as in the TA arm. Each session lasted about 60 minutes.

#### Conventional treatment

Patients allocated to the conventional treatment group (CT) were treated according to local practices at each primary care center. Treatments included watchful waiting, pharmacological and/or psychological or psycho educational therapies. CT was evaluated after four and eight weeks irrespective of the length of treatment.

### Randomization

**Figure 1:**
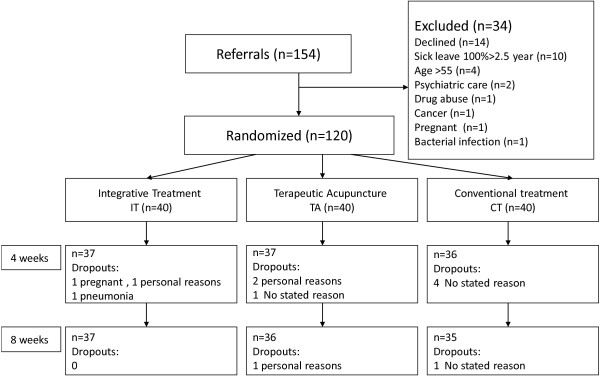
Flowchart of the patients in the study.

After baseline assessments, patients were randomly allocated to one of the three treatment regimens. One hundred and twenty cards printed with the letters A, B or C, designating treatment group, were prepared and placed in sealed, opaque envelopes by a person with no connection with the study. The envelopes were shuffled and mixed together in a box. A research nurse, blind to the aims of the study and card coding system, first mixed the envelopes again and then randomly selected envelopes from the box and thereby established the randomization sequence (Figure 1).

#### Data collection and assessment instruments

Assessments were conducted by means of mailed self-rated questionnaires at baseline and after four and eight weeks of treatment completion. Baseline questionnaires were returned at the initial visit and follow-up questionnaires were returned by mail. Primary outcomes were anxiety and depression as measured by the Hospital Anxiety and Depression Scale. The Hospital Anxiety and Depression Scale (HAD) [49] is a 14-item self-rating scale that consists of the subscales of anxiety (HADS-A) and depression (HADS-D). Ratings are made against a four-point scale (0–3) and scores are calculated by summing the ratings of the seven items comprising each subscale, where higher scores indicate more depression or anxiety. Scores between 8 and 10 have been suggested to represent possible cases of anxiety or depression and scores of 11 or above to correspond to probable cases [50]. Validity and reliability of the Swedish version of the HAD has been assessed [51]. In that study, the 2-factor structure of the original HAD was replicated; Cronbach’s alpha for HAD anxiety and depression was .84 and .82, respectively; and correlations with the Beck Depression Inventory were r = .64 for HAD anxiety and r = .71 for HAD depression.

#### Statistical analyses

Analyses were performed on an intention-to-treat basis. Descriptive statistics were used to characterize socio-demographic, clinical and outcome variables at baseline and follow up in each treatment group. Baseline between-group differences in gender and education level were assessed with the Chi2 test and age was evaluated with a one-way ANOVA. Between-group differences in HAD depression and anxiety at baseline and follow up, as well as change from baseline were assessed with the non-parametric omnibus Kruskal-Wallis test, followed by pairwise comparisons with the Mann–Whitney U test. Non-parametric methods were used due to the skewed distribution and ordinal-level of the HAD data. Scores for missing questionnaires were imputed as the treatment group mean. All tests were two-tailed and a 5% significance level was used throughout. All analyses were conducted using PASW SPSS version 18 (Chicago, Il) [52].

The clinical significance of change was assessed using effect sizes (ES), published minimal important difference (MID) estimates and the reliable change index (RCI). Effect sizes (ES) were calculated to estimate the magnitude of the within-group differences in HAD values between baseline and 8-week follow up. ES were calculated as the difference between mean values divided by the pooled standard deviation at baseline. ES magnitudes were interpreted against the criteria suggested by Cohen: trivial (0 to <0.2), small (≥0.2 to <0.5), moderate (≥0.5 to <0.8) and large (≥0.8) [53]. Published estimates of MIDs (Δ ≥ 1.5 points) were also used to assess the clinical significance of HAD anxiety and depression mean change scores [51]. Additionally, the RCI [54] was used to determine the number of patients in each group who achieved change scores that were both reliable and clinically significant. RCI analyses were conducted in two steps. Firstly, the RCI was used to assess whether the individual change was statistically significant. The RCI is defined as the change in scores divided by the standard error of the difference for the test. The standard error of difference was calculated based on the standard deviation and published test-retest reliability correlations for the HAD [55]. The cut-off for statistical significance on the RCI is 1.96, which corresponds to the 95% confidence interval (CI). Secondly, the numbers of patients with significant RCIs who had also improved from HAD possible or probable anxiety versus depression at baseline to noncases at 8-week follow-up were determined. Those patients who met both conditions were considered to have made clinically significant improvements. Between-group differences in proportions was tested with the Chi^2^ test. Only patients above the cut-off for caseness (HAD > 8) at baseline were included in these analyses.

## Results

A total of 108 (90%) of the 120 randomized participants completed eight weeks of treatment. Of these, 37 were in the IT group (86% women), 36 in the TA group (83% women) and 35 in the CT group (95% women). Ten of the 12 non-compliers completed fewer than 4 weeks of treatment (Figure 1). The treatment groups did not differ significantly at baseline with respect to age, gender or education level (Table 1) or HAD anxiety and depression scores (Table 3).

**Table 3:** HAD scores at baseline, after four weeks and after eight weeks of treatment

**HAD**	**IT (n = 40)**			**TA (n = 40)**			**CT (n = 40)**			**IT-TA-CT**	**IT-CT**	**TA-CT**	**IT-TA**
	**Mean**	**SD**	**Median**	**Mean**	**SD**	**Median**	**Mean**	**SD**	**Median**	**p-value**^**1**^	**p-value**^**2**^	**p-value**^**2**^	**p-value**^**2**^
**Anxiety**													
Baseline	9.75	5.24	9.50	11.58	4.74	12.00	11.65	4.57	12.00	0.197	0.105	0.877	0.138
4 weeks	7.54	4.34	6.00	7.97	4.18	7.97	10.18	4.16	10.19	**0.005**	**0.005**	**0.006**	0.446
8 weeks	5.40	3.99	5.00	6.94	4.14	6.47	10.35	4.43	11.00	**0.000**	**0.000**	**0.001**	0.093
**Depression**													
Baseline	7.61	3.63	7.00	7.65	4.23	8.00	7.48	4.21	8.00	0.991	0,965	0.870	0.969
4 weeks	6.12	3.41	6.00	5.38	3.71	5.38	7.44	3.59	7.42	**0.006**	**0,034**	**0.002**	0.228
8 weeks	3.81	2.73	4.00	4.11	3.04	4.00	7.47	3.66	7.47	**0.000**	**0.000**	**0.000**	0.625

HAD: Hospital Anxiety and Depression scale. ^1^Nonparametric Kruskal-Wallis test, ^2^Mann-Whitney-U’s test, p-values less than 0,05 are shown in bold.

**Figure 2:**
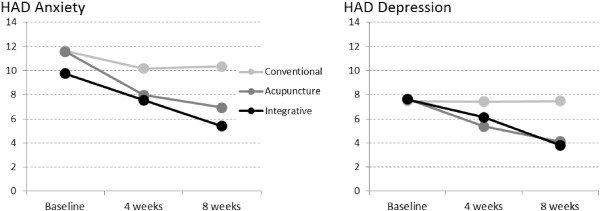
HAD anxiety and depression mean scores at baseline and after four and eight weeks of treatment.

**Figure 3:**
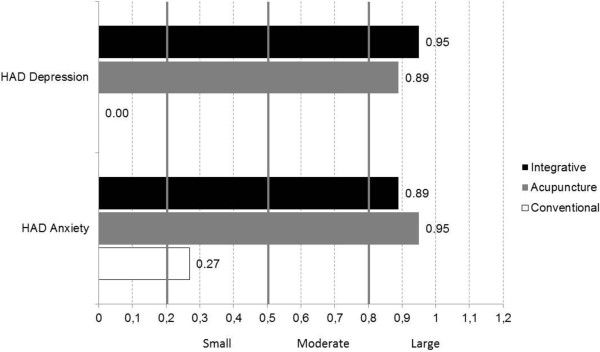
HAD anxiety and depression within-group effect sizes between baseline and eight weeks of treatment.

At both 4 and 8-weeks, between-group differences were noted for both HAD depression and HAD anxiety (Table 3). Posthoc comparisons yielded significant differences between CT versus TA and IT; however, no differences were found between TA and IT (Table 3, Figure 2). Similarly, both TA and IT improved significantly more than CT (both p < 0.001) from baseline to 8 weeks, but no differences were found between the former treatment groups. Mean HAD depression and anxiety change scores were also clinically significant (Δ > 1.5) in both TA (Δ_dep_ = 3.53; Δ_anx_ = 4.63) and IT (Δ_dep_ =3.81; Δ_anx_ = 4.34 ), but not in CT (Δ_dep_ = 0.01; Δ_anx_ = 1.30). Effect sizes were large (ES > .80) in TA (ES = .95; CI = 1.42-.49) and IT (ES = .89; CI = 1.34-42) in relation to anxiety, and large in relation to depression (ES = .89; CI = 1,34-.42 and .95; CI = 1.41-.48, respectively). In the CT group, effect sizes were small for anxiety (ES = .27; CI = .70--.18) and trivial for depression (ES = .00; CI = .44--.44) (Figure 3). Moreover, 50% of the patients in the IT group, 48% in the TA group and 10% in the CT group achieved clinically significant improvements (RCI > 1.96 + change from case to noncase) in HAD anxiety. These proportions were significantly different (chi^2^ = .003). Likewise, 53% of the patients in the IT group, 50% in the TA group and 5% in the CT group achieved clinically significant improvements in HAD depression (chi^2^ = .008).

### Dropouts

In total, 3 IT, 4 TA and 5 CT patients dropped out during the study period. All 3 IT patients dropped out before four weeks; 3 of the 4 TA patients dropped out before four weeks and one before eight weeks; and 4 of 5 CT patients dropped out before four weeks and one before eight weeks (Figure 1). Reasons for dropout were pregnancy, personal reasons and pneumonia in the IT group; personal reasons (n = 2) and no stated reason in the TA group; and no stated reason (n = 4) in the CA group.

## Discussion

This pragmatic randomized controlled study showed that both the integrative treatment and therapeutic acupuncture significantly, both statistically and clinically, reduced anxiety and depression compared to usual primary care in patients with psychological distress. Moreover, about half of the patients in the integrative and acupuncture groups with initial depression or anxiety achieved significant improvements after treatment. On the other hand, conventional treatment approaches commonly used in Swedish primary care settings yielded only small and clinically non-significant effects, and only 10% clinically improved in anxiety and 5% in depression. Our results thus corroborate earlier findings indicating the efficacy of acupuncture in reducing anxiety, e.g., [56] and depression, e.g., [37] but fail to support our hypothesis that the integrative intervention would further enhance the effectiveness of therapeutic acupuncture. Some support for the added benefit of the integrative treatment was evidenced by slightly larger effect sizes in relation to depression; however, the reverse held true in the case of anxiety.

The failure to observe substantial added benefits of the integrative treatment may owe to a number of reasons. Firstly, there may have been contamination between treatments, as acupuncture therapy was performed by the same experienced, certified acupuncturist in both groups. Although this crossed design has the advantage of controlling for therapist effects and is commonly used in psychotherapy research for this purpose, it has the disadvantage that distinctions between performed treatments may be blurred [57]. Hence, the effects of acupuncture per se may have been augmented. By the same token, however, the effects of the integrative treatment may have been affected by treatment allegiance bias. Secondly, the study may have been underpowered. At the time this study was planned no published studies had reported estimates for the Hospital Anxiety and Depression Scale upon which to base power calculations. Since that time, Puhan et al. [58] have published minimal important differences for the HAD. Based on these estimates we would have required about 60 patients per treatment, instead of the 40 included here. Hence, larger studies that carefully demarcate treatment arms are needed to evaluate head-to-head the added value of the adjunctive therapy over and above acupuncture alone.

The patient sample was heterogeneous with respect to diagnosis, which reflects the diversity of mental and somatic health problems found in primary care. It is important to note, however, that patients were not necessarily referred for treatment for their primary diagnosis, but rather for a suspected underlying psychological distress. Hence, patients with, for example, medically unexplained syndromes, such as IBS and fibromyalgia, were not referred for treatment for their somatic symptoms, but rather for the distress associated with their condition. In this respect the sample was homogeneous. In line with this and in order to standardize acupuncture treatment in the study, relatively fixed restorative and sedative acupoints were selected for all patients in the integrative and acupuncture groups. This is at variance with principles of TCM, which espouse individualized selection of acupoints, and may in fact have dampened the effects of acupuncture.

On the other hand, levels of psychological distress in the sample varied greatly from severe (as indicated by a diagnosis of clinical depression and/or panic or anxiety disorder, or a “probable case” classification according to conventional HAD criteria) to very mild symptoms of depression and/or anxiety. In fact, baseline HAD anxiety scores indicated that nearly 25% of the sample had at most mild symptoms (non-cases), 25% had moderate symptoms (possible cases) and 50% had symptoms suggesting probable clinical levels of anxiety. Corresponding proportions for HAD depression were 47% for mild symptoms, 33% with moderate and only 20% with severe symptoms. This variation may reflect the acknowledged difficulties general practitioners have in assessing the severity of mental health problems in their patients [22,23] or it may reflect a recognition of the prophylactic importance of providing treatment even to patients with mild to moderate symptoms ref. A striking result in this study was that 82% of those patients with moderate (possible case) to severe depression (probable case) and 63% with corresponding levels of anxiety at baseline were classified as non-cases after acupuncture treatment (with or without adjuvant integrative treatment). In contrast, conventional treatment resulted in only a 10% increase in anxiety and depression non-cases.

Attrition rates were low in all three treatment groups, suggesting good acceptance of the provided treatments. Only 3 patients in the integrative treatment group, 4 in the acupuncture group and 5 in the conventional treatment group dropped out for various reasons during the study period. Missing HAD scores for these patients were imputed as the group mean for each respective treatment group. Because these patients had generally worse baseline HAD values than group averages, the use of simple mean imputation may thus have affected outcomes slightly in favor of conventional treatment; however, sensitivity analyses showed no such effects.

The sample consisted predominantly of women (84%). This probably reflects the fact that women are generally more likely to discuss mental health problems with their physician than are men [59] and therefore are more likely to be offered treatment. The small number of men in the study precluded meaningful analyses of gender differences in relation to treatment outcomes and our results may therefore be considered to apply primarily to women with psychological distress.

A limitation of the study was the short follow-up period; hence, we are currently examining the intermediate term effects (6 months) of the treatments. Only four primary care centers participated in the study and we cannot be sure that treatment options offered at these centers are representative of those provided at primary care in Sweden.

## Conclusion

The integrative treatment and therapeutic acupuncture significantly, both statistically and clinically, reduced symptoms of anxiety and depression in primary care patients with psychological distress; however, no differences were found between these modalities. On the other hand, conventional primary care treatment yielded only small and clinically non-significant effects. Larger and longer term studies are needed to evaluate the added value of the integrative treatment over therapeutic acupuncture alone.

### Clinical implications

Although mental health problems are prevalent, their detection, diagnosis and treatment are inadequate in primary care setting [60]. Our results indicate that acupuncture and integrative treatment may be useful and acceptable adjunct regimens for patients identified with symptoms of psychological distress in such settings. More research is needed, however, to examine if the integrative treatment offers additional clinical benefit over acupuncture alone.

## Competing interests

The authors declare that they have no competing interests.

## Authors’ contributions

TA and CT contributed to planning the study. TA collected data. TA and CT analysed the data. TA, BM and CT authors contributed to the writing of the manuscript. All authors read and approved the final manuscript.

## Pre-publication history

The pre-publication history for this paper can be accessed here:

http://www.biomedcentral.com/1472-6882/13/308/prepub
